# Tuberculous Meningitis With Persistent Hiccups: A Case Report

**DOI:** 10.7759/cureus.92526

**Published:** 2025-09-17

**Authors:** Satoshi Inaba, Sonoko Matsuyama, Masayuki Oda, Atsushi Kawashima

**Affiliations:** 1 Department of General Internal Medicine, Fukuchiyama City Hospital, Fukuchiyama, JPN; 2 Department of General Medicine and Community Healthcare, Kyoto Prefectural University of Medicine, Kyoto, JPN

**Keywords:** aseptic meningitis, diagnostic uncertainty, hiccups, nonspecific symptoms, tuberculosis meningitis

## Abstract

A 69-year-old Japanese male presented with atypical symptoms of tuberculous meningitis (TBM), including intermittent left temporal headache and persistent hiccups that were unresponsive to initial therapies. Despite comprehensive imaging, no definitive intracranial lesions were identified. His persistent hiccups, along with typical meningitis symptoms, highlighted the diagnostic challenges of TBM, particularly in the context of negative microbiological tests. Early empirical treatment, initiated based on clinical suspicion and elevated cerebrospinal fluid (CSF) adenosine deaminase levels, led to clinical improvement and supported a diagnosis of TBM. This case underscores the importance of considering TBM in patients with unusual symptoms, such as persistent hiccups, to prevent delays in diagnosis and treatment.

## Introduction

Tuberculous meningitis (TBM) is the most severe form of extrapulmonary tuberculosis, with devastating consequences. TBM is a subacute meningoencephalitis caused by Mycobacterium tuberculosis, classically presenting with fever, headache, and altered mental status; however, this triad is often incomplete at first presentation [1]. Atypical early symptoms, such as persistent hiccups, are under-recognized and may further obscure diagnosis [2]. Although considered relatively rare in developed countries, it affects 1-5% of all tuberculosis patients and carries high rates of mortality and morbidity; approximately half of the patients either succumb to the disease or suffer from long-term neurological sequelae [3]. The diagnosis of TBM, however, remains a significant clinical challenge. The initial symptoms are often nonspecific, including headache, nausea, loss of appetite, and weight loss, which can mimic other more common conditions [1]. Furthermore, a definitive diagnosis is frequently elusive. Many patients have no prior history of tuberculosis, and only about half present with concomitant extrameningeal tuberculous lesions [4].

The cornerstone of diagnosis, cerebrospinal fluid (CSF) analysis, is fraught with difficulties. Direct detection of Mycobacterium tuberculosis is challenging due to the paucibacillary nature of the disease. The sensitivity of CSF smear microscopy (Ziehl-Neelsen staining) is as low as 20-40%, and while CSF culture is more sensitive (40-80%), it can take several weeks to yield results, delaying critical treatment initiation [5-7]. Atypical clinical presentations can further complicate and delay timely diagnosis. Beyond the individual diagnostic challenge, TBM continues to impose substantial global mortality and disability, and delays in diagnosis and treatment are strong predictors of poor outcomes [3]. Because early clinical features are frequently nonspecific [2], improving recognition of atypical presentations, such as persistent hiccups, could help shorten time to therapy and mitigate the impact of diagnostic delay [1,3].

We report a case of TBM in a 69-year-old male whose primary atypical symptom was persistent hiccups, highlighting this diagnostic dilemma and underscoring the importance of high clinical suspicion.

## Case presentation

A 69-year-old Japanese male with no significant past medical history presented with a one-week history of intermittent left temporal headache. The head computed tomography (CT) was unremarkable, and zoster sine herpete was initially suspected. The headache progressed, becoming constant in the left temporal-occipital region. He developed a reduced stride, bowlegged gait, unsteadiness, and dysarthria, prompting an emergency department visit five days later.

On arrival, his Glasgow Coma Scale score was E4V4M6. His vital signs were as follows: blood pressure 131/92 mmHg, heart rate 106 bpm, respiratory rate 36 breaths/min, temperature 39.0 °C, and oxygen saturation 96% on ambient air. Physical exam showed no rash. Neck stiffness was noted. For meningeal irritation signs, neck flexion was positive. Neurological examination revealed meningeal signs and gait unsteadiness, but no focal neurological deficits or cerebellar ataxia.

Blood tests revealed mild abnormalities (Table 1). Suspecting meningitis, a lumbar puncture was performed (Table 2). Gram stain of CSF was negative. CSF was tested for culture, adenosine deaminase (ADA), cryptococcal antigen, and polymerase chain reaction (PCR) for herpes simplex virus and varicella zoster virus. Urgent head and trunk computed tomography (CT) and magnetic resonance imaging (MRI) revealed no notable findings.

**Table 1:** Laboratory findings of blood tests at the time of admission

Test	Result	Normal Range
White Blood Cell (WBC)	5.61 x10^3^/uL	3.50 to 8.50 x10^3^/uL
Neutrophil	76.9 %	40 to 71 %
Lymphocyte	14.3 %	20 to 50 %
Monocyte	8.4 %	0 to 8 %
Eosinophil	0.0 %	0 to 6 %
Basophil	0.4 %	0 to 2 %
Hemoglobin (Hb)	15.3 g/dL	14.0 to 17.7 g/dL
Platelet	170 x10^3^/uL	140 to 340 x10^3^/uL
Total Protein (TP)	7.2 g/dL	6.7 to 8.3 g/dL
Albumin	4.2 g/dL	4.0 to 5.0 g/dL
Total Bilirubin (T-Bil)	0.6 mg/dL	0.2 to 1.0 mg/dL
Aspartate Aminotransferase (AST)	22 IU/L	10 to 40 IU/L
Alanine Aminotransferase (ALT)	23 IU/L	5 to 40 IU/L
Lactate Dehydrogenase (LDH)	205 IU/L	115 to 245 IU/L
Alkaline Phosphatase (ALP)	131 IU/L	38 to 113 IU/L
Gamma-Glutamyltransferase (γGTP)	41 IU/L	0 to 70 IU/L
Creatine Kinase (CK)	264 IU/L	57 to 197 IU/L
Blood Urea Nitrogen (BUN)	23 mg/dl	6 to 20 mg/dL
Serum Creatinine	0.74 mg/dl	0.61 to 1.04 mg/dL
Serum Sodium	131 mmol/L	136 to 147 mmol/L
Serum Potassium	4.0 mmol/L	3.6 to 5.0 mmol/L
Serum Chloride	95 mmol/L	98 to 109 mmol/L
Serum Calcium	8.3 mg/dL	8.7 to 10.1 mg/dL
C-reactive Protein (CRP)	0.05 mg/dL	0 to 0.30 mg/dL
Blood Glucose	115 mg/dL	70 to 110 mg/dL

**Table 2:** Sequential cerebrospinal fluid test results

Test	Result			Normal Range
	Day of Admission	Day 5	Day 17	
Cell Count	124 /uL	288 /uL	137 /uL	0 to 35 /uL
Mononuclear Cells	87 %	98 %	99 %	N/A
Polymorphonuclear Cells	13 %	2 %	1 %	N/A
Total Protein	188 mg/dL	78 mg/dL	84 mg/dL	10 to 40 mg/dL
Glucose	47 mg/dL	51 mg/dL	54 mg/dL	40 to 75 mg/dL
Chloride	117 mmol/L	119 mmol/L	119 mmol/L	120 to 125 mmol/L
Xantocromia	Negative	Negative	Negative	Negative
Adenosine Deaminase (ADA)	17.7 IU/L	17.4 IU/L	13.2 IU/L	N/A

He was admitted with suspected meningitis and started on empiric broad-spectrum antibiotics and acyclovir. Shortly after admission, he developed urinary retention requiring catheterization, which we considered meningitis-retention syndrome (MRS) secondary to meningitis. Over the next few days, empiric treatments for bacterial and viral meningitis were discontinued because the clinical course was not supportive and testing was negative, including CSF and blood bacterial cultures and CSF PCR for herpes simplex virus and varicella zoster virus. However, he developed fever and persistent hiccups unresponsive to standard treatments, including metoclopramide, lansoprazole, and baclofen. On day 4, a contrast-enhanced brain MRI (gadolinium) was performed to evaluate for medullary and area postrema involvement and other alternative CNS causes of hiccups, including brainstem infarct, and it showed no significant abnormalities. Repeat CSF analysis on day 5 showed worsening pleocytosis. Review of the initial CSF revealed elevated ADA (17.7 U/L), which remained high (17.4 U/L) on day 5. Tuberculous meningitis was suspected, and anti-tuberculosis therapy (rifampicin 600 mg, isoniazid 300 mg, pyrazinamide 1,500 mg, and ethambutol 1,000 mg) was initiated on day 8. Adjunctive corticosteroids were not administered due to the absence of definitive imaging findings (e.g., tuberculomas) and the uncertainty of the diagnosis.

Three days after starting treatment, the fever and hiccups improved. By day 17, repeat CSF showed improvement (Table 2). CSF smear, culture, and PCR for acid-fast bacilli remained negative. An interferon-gamma release assay (T-SPOT® TB, Oxford Immunotec Ltd., UK; IGRA), performed on day 10, was also negative. No pulmonary or extrapulmonary tuberculosis lesions were detected on trunk CT, and there were no findings suggestive of prior tuberculosis (e.g., apical scarring or parenchymal/pleural calcifications, or calcified mediastinal/hilar lymph nodes). Despite the lack of microbiologic confirmation, the symptom resolution and decline in CSF ADA supported a clinical diagnosis of TBM (Table 2). Evaluation for an underlying immunodeficiency was unremarkable; the HIV test was negative, HbA1c was 6.3%, and there was no history of increased susceptibility to infections. Furthermore, there was no personal or known sick contact, and the source of infection was unclear.

**Figure 1:**
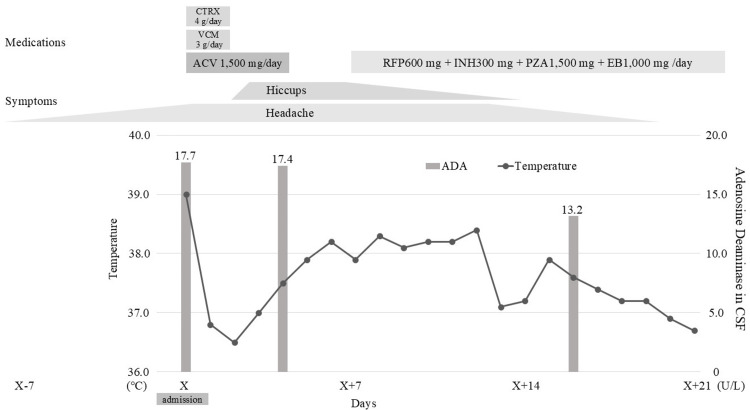
Clinical course of the patient He initially presented with a headache and was started on meningitis treatment. Despite this, he developed hiccups. CSF analysis revealed elevated levels of ADA, leading to the initiation of a four-drug anti-tuberculosis regimen (RFP 600 mg, INH 300 mg, PZA 1,500 mg, and EB 1,000 mg per day). Over time, his fever and symptoms gradually improved. Re-evaluation of CSF ADA levels after the initiation of anti-tuberculosis treatment showed a declining trend. Based on the clinical course, a diagnosis of tuberculous meningitis was made. He completed a total of nine months of treatment after discharge.

His condition gradually improved, and he resumed pre-morbid activities of daily living, allowing discharge on day 21 (Figure 1). As the fever subsided and hiccups diminished after initiation of anti-tuberculosis therapy, dysarthria and gait unsteadiness improved in parallel, with near-complete resolution by discharge. Because clinical symptoms and CSF parameters improved on anti-tuberculosis therapy alone, adjunctive corticosteroids were not reconsidered. He continued outpatient care without recurrence of symptoms or adverse effects of anti-TB medications. After completing nine months of anti-TB therapy, a repeat IGRA was performed to reassess a potential false-negative result during early infection; it remained negative. We did not repeat the lumbar puncture at treatment completion because the procedure is invasive, and the patient had fully recovered clinically.

## Discussion

TBM is considered a relatively rare disease in developed countries, but it occurs in 1-5% of tuberculosis patients and is a severe condition with about half of the patients either dying or suffering from long-term sequelae [3]. Most cases have no history of tuberculosis, and only about half of the patients have extrameningeal tuberculosis lesions [4]. Symptoms are often nonspecific such as headache, nausea, loss of appetite, and weight loss [1]. The initial differential diagnosis for a patient presenting with headache, fever, and meningeal signs is broad and includes more common conditions such as bacterial and viral meningitis, as well as other critical pathologies like nontraumatic intracranial hemorrhage. Even rarer entities, such as neoplastic processes presenting with diffuse meningeal nodules [8], must be considered. In this case, the absence of blood on neuroimaging, the lack of mass lesions or nodular enhancement, and the progressive, subacute course helped narrow the focus to inflammatory and infectious etiologies.

CSF examination is crucial for diagnosis, but it is often difficult to detect Mycobacterium tuberculosis in CSF, making microbiological diagnosis challenging [5]. The sensitivity of Ziehl-Neelsen staining and culture of CSF is limited, reported at 20-40% and 40-80%, respectively [6,7]. A CSF ADA level of >10 U/L is considered a helpful diagnostic marker, though specificity is limited [9]. Given these limitations, it is likely that many cases remain undiagnosed [10]. In this case, the diagnosis was supported by persistently elevated CSF ADA levels and clinical improvement following anti-tuberculosis therapy, despite negative microbiological findings. We also note mild hyponatremia (Na 131 mmol/L) at presentation. Although not specific, hyponatremia is reported frequently in TBM and may offer a supportive context when TBM is suspected [11]. In addition, this patient had two negative IGRAs. The negative IGRA, while challenging the diagnosis, is not unprecedented in extrapulmonary TB and may be due to anergy or the localized nature of the infection. Accordingly, IGRA negativity should not be used to exclude TBM when clinical suspicion is high [12].

A distinctive feature of this case was persistent hiccups. The hiccup center is located in the dorsal medulla oblongata [13], and lesions in this area are known to cause hiccups. Hiccups have been reported in meningitis, including TBM [2], but their prevalence remains unclear. A single-center retrospective study reported hiccups in a subset of TBM patients [2]; however, it remains an under-recognized presentation that should raise suspicion. The mechanism may involve inflammatory extension to the dorsal medulla due to meningitis. However, in this case, contrast-enhanced MRI showed no abnormal meningeal enhancement, and trunk CT revealed no intrathoracic or upper abdominal anatomical abnormalities that could account for the hiccups, and the exact cause remained unknown.

Ultimately, TBM was diagnosed based on the clinical picture of aseptic meningitis with persistent hiccups, elevated CSF ADA, and a favorable response to treatment. Although the diagnosis remained presumptive in the absence of definitive microbiological or radiological evidence, the improvement in symptoms and CSF markers supported the clinical judgment. Diagnosing TBM is especially difficult in the absence of typical imaging findings or cranial nerve involvement such as abducens nerve palsy. In this case, nonspecific symptoms--headache, fever, hiccups, and urinary retention--complicated the diagnostic process. In practice, persistent hiccups with meningitic symptoms and aseptic CSF should prompt consideration of TBM even with unrevealing imaging; CSF ADA >10 U/L can support presumptive diagnosis when direct tests are negative [9]; early empirical therapy may be warranted when suspicion is high; and autonomic signs, such as urinary retention, may accompany meningitis.

## Conclusions

Diagnosing tuberculous meningitis is challenging, particularly when microbiological evidence is lacking and imaging findings are unremarkable. A high index of suspicion should be maintained even in cases with atypical symptoms such as persistent hiccups. Early empirical treatment based on clinical assessment may prevent delayed diagnosis and improve outcomes. Prospective documentation of atypical TBM symptoms, including persistent hiccups, may improve recognition and inform future guidelines.
